# Prevalence of caregiving and high caregiving strain among late-career medical school faculty members: workforce, policy, and faculty development implications

**DOI:** 10.1186/s12960-021-00582-3

**Published:** 2021-03-19

**Authors:** Kimberly A. Skarupski, David L. Roth, Samuel C. Durso

**Affiliations:** 1 Division of Geriatric Medicine and Gerontology, School of Medicine, Epidemiology, Bloomberg School of Public Health, Johns Hopkins University, Baltimore, MD USA; 2 Division of Geriatric Medicine and Gerontology, School of Medicine, Biostatistics, Bloomberg School of Public Health; and Director, Center on Aging and Health, Johns Hopkins University, Baltimore, MD USA; 3 Division of Geriatric Medicine and Gerontology and Executive Vice Chair, Department of Medicine, Johns Hopkins Bayview Medical Center, Baltimore, MD USA

**Keywords:** Caregiving, Faculty development, Academia, Workforce

## Abstract

**Background:**

Nearly one-third of medical school faculty members are age 55 + . As our population ages, the prevalence of family caregiving is increasing, yet we know very little about the caregiving experiences of aging faculty members in academic medicine. Faculty caregiving responsibilities coupled with projected physician shortages will likely impact the future academic medical workforce. We examined the prevalence of caregiving, concomitant caregiving strain, general well-being, and thoughts about retirement for medical school faculty members age 55 and older.

**Methods:**

We analyzed data from a survey of 2,126 full-time medical school faculty 55 + years of age conducted in 2017. Chi-square tests of independence and independent samples t-tests were used to examine statistical differences between subgroups.

**Results:**

Of the 5,204 faculty members invited to complete the parent survey, 40.8% participated (*N* = 2126). Most were male (1425; 67.2%), White (1841; 88.3%), and married/partnered (1803; 85.5%). The mean age was 62.3 years. Of this sample, 19.0% (*n* = 396) reported providing care on an on-going basis to a family member, friend, or neighbor with a chronic illness or disability, including 22.4% (*n* = 154) of the female respondents and 17.3% (*n* = 242) of the male respondents. Among the caregiving faculty members, 90.2% reported experiencing some or a lot of mental or emotional strain from caregiving. Caregivers gave lower ratings of health, social and emotional support, and quality of life, but greater comfort in religion or spirituality than non-caregivers. Both caregiving and non-caregiving faculty members estimated retiring from full-time employment at age 67.8, on average.

**Conclusion:**

These data highlight caregiving responsibilities and significant concomitant mental or emotional strain of a significant proportion of U.S. medical schools’ rapidly aging workforce. Human resource and faculty development leaders in academia should strategically invest in policies, programs, and resources to meet these growing workforce needs.

## Background

As populations age, roles and responsibilities of aging employees, specifically caregiving roles, change. Concomitantly, employers must recognize these work–life integration changes and challenges. In 2019, adults age 55 or older constituted 29.4% of the entire U.S. population [1] and among *employees*, the Bureau of Labor Statistics estimated that workers age 55 and older will rise to 24.8% by 2026 [2]. In medical schools, there is no mandatory retirement for faculty [3] and coupled with the anticipated need for physicians due to the estimated shortage in the next ten years [4], academic medical faculty may remain in the workforce longer than in previous decades.

Indeed, we are witnessing the aging of our medical schools’ faculty populations. In 2019, the average age of all full-time medical school faculty members in the U.S. (*N* = 178,871) was 49.1 years [5] compared to 44.7 years in 1987 [6]. Faculty members who were age 55 or older comprised 31% of the full-time medical school faculty population in 2019 [5] compared to 19% in 1987 [6]. For comparison purposes, among our institution’s school of medicine, University-paid, full-time employees (*N* = 9011), 25.4% of non-faculty employees (*N* = 6148) were age 55 and older compared to 28.3% of faculty (*N* = 2863).

One reason for our rapidly aging population is that life expectancy has been increasing over the past few decades. An average American woman at age 65 is now expected to live another 20.5 years and a man age 65, another 18 years [7]. Correspondingly, as populations age and live longer, caregiving needs inevitably rise. Thus, faculty members and their care recipients are living longer, necessitating even more care. Unfortunately, lower fertility rates have also reduced the number of available family member to provide or assist with caregiving [8]; hence, our future aging faculty members may have an even greater likelihood of serving as caregivers to their spouses or other family members.

In the gerontologic literature, it is well known that family and unpaid caregivers provide the majority of care for older adults [9, 10]. According to the 2015 Bureau of Labor Statistics, it was estimated that during 2013–2014, 16.1% (40.4 million) of the U.S. civilian noninstitutionalized population age 15 and older provided unpaid care to someone age 65 or older. Among those providing care, 47% were employed full-time [11]. Despite there being a literature base aimed at mitigating the effects of caregiving challenges on *early-career* faculty members’ (specifically, female) careers [12], we know very little about caregiving challenges among *late-career* faculty members [13] and even less about how caregiving responsibilities impact academicians’ career and retirement decisions.

Caregiving and caregiving strain in particular, are known to have negative effects on caregiver physical, mental, and social health [14–23]. For example, in the Roth et al. study of 43,099 community-dwelling adults (average age 65.5, SD = 9.8), 12.0% of participants were caregivers; they averaged 63.5 years of age and 49% reported some strain, 18% reported a lot of strain, and 33% reported no strain. Those who reported high caregiving strain had poorer quality of life than less-strained caregivers and non-caregivers [14]. A follow-up study 10 years later showed a steadily increasing prevalence of caregiving and caregiving strain. Roth et al. found that 12.4% of the community-dwelling respondents (*N* = 13,270) were caregivers and 52% reported some strain, 19% reported a lot of strain, and 29% reported no strain [24].

Unsurprisingly, it has also been established that caregiving and caregiving burden also affect employee wellness and productivity. In their survey of 18,120 federal employees, Buffardi et al. found that the primary negative effects of *child care* were on leave benefits and work–family balance, whereas *caregiving for older adults* was associated with lower levels of satisfaction with perceived organizational support, pay, leave benefits, and work–family balance [25]. Gaugler et al. analyzed data from a large health-care plan employer (*N* = 880) and reported that compared to their non-caregiving employee counterparts, employed caregivers were more likely to indicate poorer physical and mental health [26].

In addition to deleterious health consequences, the caregiving experience differs by gender. Caregiving is often characterized as a role predominantly taken by women [11], but this gender difference may be diminishing. Indeed, Wolff et al. found in longitudinal national samples, that more men are taking on family caregiving roles in more recent surveys (31.8% in 1999 to 36.3% in 2015), especially for non-dementia caregiving [17]. However, there has been no published literature exploring the prevalence of caregiving and differences by gender among late-career faculty members.

In the analyses presented here, we describe the previously unknown prevalence of caregiving among late-career faculty members in academic medicine by gender, associated caregiving strain, cursory measures of health and well-being, and thoughts about retirement. Second, we speculate that future demographic aging and caregiving trends have workforce planning implications and will necessitate increased attention to retirement planning in academia.

## Methods

This project is a secondary data analysis from a survey of 2,126 full-time faculty members age 55 and older at 14 U.S. LCME-accredited medical schools conducted in May–September, 2017 [27, 28]. The goal of the original project was to gain a better understanding of full-time faculty members in academic medicine who were age 55 and to describe their work–life expectations.

For the survey, we employed a purposive sampling strategy and identified 14 medical schools to maximize representativeness of the US LCME-accredited medical school population based on: geographic region [Northeast = 3 (21%), Central = 4 (29%), South = 4 (29%), West = 3 (21%); public [7 (50%)] or private [7 (50%)] ownership; community-based [2 (14%)] or non-community-based status [12 (86%)]; financial relationship with a parent university; and number of full-time faculty. We chose to conduct a sample survey, as opposed to a population survey of all 155 US LCME-accredited medical schools, to reduce institutional survey burden, especially given the routine, institution-wide data reporting requirements, as well as for study expediency. The purposive sampling strategy is the AAMC’s preferred sampling strategy. Upon agreeing to participate in the survey, each institution’s faculty affairs or faculty development dean sent descriptive emails to faculty members age 55 or older inviting them to participate in the survey.

We obtained standard sociodemographic data via self-report, including: sex; age; race; marital status; number of people in the household; and personal finances. Actual age was collected and then we reported age categories. For race-ethnicity, we aggregated the original eight categories (American Indian/Alaskan Native; Asian; Black or African; Hispanic, Latino, Spanish; Multiracial/ethnic; Native Hawaiian or other Pacific Islander; Other; and White) into two groups, majority (White and Asian) and minority (Black, Hispanic, Others). For marital status, we aggregated the responses into two groups, ‘married (or partnered)’ or ‘single’. The single category included separated, divorced, widowed, and never married. We asked for the number of people living in the household and aggregated the responses into 1, 2, and 3 or more. Respondents were also asked to indicate if their personal finances were: sufficient; not sufficient; or unsure.

To minimize overall survey burden, we constrained our caregiving items to three validated questions adapted from previous research [14–16, 24]: (1) Are you currently providing care on an on-going basis to a family member, friend, or neighbor with a chronic illness or disability (Yes, No, prefer not to answer); (2) Does the person for whom you provide on-going care currently live with you? (Yes, No, prefer not to answer); and (3) How much of a mental or emotional strain is it on you to provide this care? (No strain, Some strain, A lot of strain, prefer not to answer).

As a rudimentary assessment of general well-being, we asked six, single-item standard questions: (1) In general, would you say your health is excellent, very good, good, fair, or poor?; (2) During the past week, for much of the time I felt depressed (Yes, No); (3) How often do you get the social and emotional support you need? (Always, Usually, Sometimes, Rarely, Never); (4) How would you rate your quality of life? (Very good, Good, Neither good nor poor, Poor, Very poor); and (5) I find comfort in my religion or spirituality many times a day, every day, most days, some days, once in awhile, never/almost never, not applicable. As reported previously [27], we also asked questions about retirement and included one of those questions in the current analyses—Have you thought about or begun to think about retiring from full-time employment in academic medicine? (Yes, I plan to or think I might retire from full-time employment at age: ___; I am not sure about when I might retire from full-time employment, but it will likely be in the next 5 years; I am not sure about when I might retire from full-time employment, but it will likely be in the next 10 years; No, I haven’t yet begun to think about retiring from full-time employment [please specify why not]).

The study was approved by the American Institutes for Research’s institutional review board. Survey respondents were informed—both in the introductory email from their institution’s faculty affairs and development office leaders and in the survey instructions—that the survey was anonymous, that participation was voluntary, and that they could skip any question.

We tabulated the univariate statistics for the total sample (*N* = 2126) and conducted the Chi-square test of independence to compare proportionate differences and the independent samples t-tests for statistical differences key subgroups of participants: those providing care vs. those not providing care; and, among the caregivers, between males and females. We analyzed data using The Statistical Package for the Social Sciences (SPSS) for Windows (version 24, Chicago, Illinois).

## Results

The total number of full-time faculty 55 or older in the 14 participating institutions surveyed was 5,204. Of these, 2,126 faculty members (40.8%) responded. The majority of the survey respondents were male (67.2%) and the average age of the survey respondents was 62.3 years (age range = 55—88) (Table 1). Most respondents were in the majority (White, Asian) race/ethnic group (94.6%) and married or partnered (85.5%). More than half (61.1%) lived in a two person household and 27.9% reported three or more people in the household. Nearly three-quarters (70.6%) reported that their finances were sufficient.

**Table 1:** Characteristics of sample (*N* = 2126)

Characteristics	Total	Providing care? (*N* = 2086)			Caregivers (*N* = 396)		
	Number (%)	Yes (*n* = 396) [19.0%]	No (*n* = 1690) [81.0%]	*p value*	Males (*n* = 242) [61.1%)	Females (*n* = 154) [38.9%]	*p value*
Sex Male Female	1425 (67.2) 697 (32.8)	242 (17.3) 154 (22.4)	1153 (82.7) 534 (77.6)	.006	–	–	
Does the care recipient live with you? Yes No	–	115 (29.5) 275 (70.5)	–	–	76 (31.7) 164 (68.3)	39 (26.0) 111 (74.0)	.232
How much of a mental or emotional strain is it on you to provide this care? A lot of strain Some strain No strain	–	84 (21.6) 266 (68.6) 38 (9.8)	–	–	52 (21.8) 161 (67.4) 26 (10.9)	32 (21.5) 105 (70.5) 12 (8.1)	.644
Age categories 55–59 60–64 65–69 70 +	753 (36.0) 738 (35.3) 389 (18.6) 209 (10.0)	152 (39.2) 155 (39.9) 59 (15.2) 22 (5.7)	584 (35.1) 569 (34.2) 325 (19.6) 184 (11.1)	.001	78 (33.1) 92 (39.0) 46 (19.5) 20 (8.5)	74 (48.7) 63 (41.4) 13 (8.6) 2 (1.3)	< .001
Race/ethnicity Majority (White, Asian) Minority (Black, Hispanic, others)	1972 (94.6) 113 (5.4)	362 (91.9) 32 (8.1)	1580 (95.4) 77 (4.6)	.006	222 (92.1) 19 (7.9)	140 (91.5) 13 (8.5)	.828
Marital status Married or partnered Single (separ., divorced, widowed, never married)	1803 (85.5) 307 (14.5)	339 (86.7) 52 (13.3)	1431 (85.1) 251 (14.9)	.413	229 (95.4) 11 (4.6)	110 (72.8) 41 (27.2)	< .001
Number of people in household 1 2 3 + Mean (SD)	228 (11.0) 1270 (61.1) 580 (27.9) 2.3 (0.9)	34 (8.7) 200 (51.3) 156 (40.0) 2.5 (1.0)	191 (11.6) 1048 (63.4) 414 (25.0) 2.3 (0.9)	< .001 < .001	9 (3.8) 130 (54.2) 101 (42.1) 2.6 (1.0)	25 (16.7) 70 (46.7) 55 (36.7) 2.3 (1.0)	< .001 .008
Personal finances Finances sufficient Finances not sufficient Unsure	1490 (70.6) 253 (12.0) 367 (17.4)	249 (63.0) 65 (16.5) 81 (20.5)	1224 (73.0) 180 (10.7) 272 (16.2)	< .001	158 (65.3) 36 (14.9) 48 (19.8)	91 (59.5) 29 (19.0) 33 (21.6)	.452
In general, would you say your health is? Excellent Very good Good Fair or poor	892 (43.8) 792 (38.9) 287 (14.1) 65 (3.2)	145 (37.7) 153 (39.7) 68 (17.7) 19 (4.9)	734 (45.4) 622 (38.5) 216 (13.4) 43 (2.7)	.003	86 (36.4) 101 (42.8) 34 (14.4) 15 (6.4)	59 (39.6) 52 (34.9) 34 (22.8) 4 (2.7)	.050
During the past week, for much of the time I felt depressed Yes No	156 (7.7) 1887 (92.3)	36 (9.4) 349 (90.6)	116 (7.2) 1500 (92.8)	.148	19 (8.1) 216 (91.9)	17 (11.3) 133 (88.7)	.286
How often do you get the social and emotional support you need? Always Usually Sometimes Rarely or never	496 (24.4) 1067 (52.4) 347 (17.1) 125 (6.1)	62 (16.1) 202 (52.6) 92 (24.0) 28 (7.3)	424 (26.2) 852 (52.7) 247 (15.3) 93 (5.8)	< .001	45 (19.1) 118 (50.2) 57 (24.3) 15 (6.4)	17 (11.4) 84 (56.4) 35 (23.5) 13 (8.7)	.191
How would you rate your quality of life? Very good Good Neither good nor poor Poor or very poor	1216 (59.7) 682 (33.5) 113 (5.5) 26 (1.3)	189 (49.1) 154 (40.0) 34 (8.8) 8 (2.1)	1005 (62.2) 522 (32.3) 74 (4.6) 16 (1.0)	< .001	121 (51.5) 92 (39.1) 21 (8.9) 1 (0.4)	68 (45.3) 62 (41.3) 13 (8.7) 7 (4.7)	.033
I find comfort in my religion or spirituality Many times a day Every day Most days Some days Once in a while or never/almost never N/A	203 (10.0) 326 (16.0) 271 (13.3) 236 (11.6) 423 (20.8) 579 (28.4)	43 (11.2) 82 (21.4) 46 (12.0) 54 (14.1) 70 (18.2) 89 (23.2)	157 (9.7) 235 (14.5) 222 (13.7) 180 (11.1) 345 (21.3) 482 (29.7)	.002	24 (10.2) 50 (21.2) 30 (12.7) 32 (13.6) 50 (21.2) 50 (21.2	19 (12.8) 32 (21.6) 16 (10.8) 22 (14.9) 20 (13.5) 39 (26.4)	.421
Have you thought about or begun to think about retiring from full-time employment in academic medicine? Yes, I plan to or think I might retire from full-time employment at age (N = 856), mean (SD) Unsure, likely in the next 5 years Unsure, likely in the next 10 years No	915 (45.2) 397 (19.6) 500 (24.7) 213 (10.5) 67.8 (4.3)	174 (45.4) 72 (18.8) 93 (24.3) 44 (11.5) 67.8 (4.7)	733 (45.6) 319 (19.9) 395 (24.6) 159 (9.9) 67.8 (4.2)	.812 .976	113 (48.5) 39 (16.7) 57 (24.5) 24 (10.3) 68.7 (4.9)	61 (40.7) 33 (22.0) 36 (24.0) 20 (13.3) 66.2 (3.9)	.346 .001

The majority of respondents reported that in general, their health was either excellent (43.8%) or very good (38.9%). The majority (92.3%) reported that they did not feel depressed during much of the time in the past week. The majority reported getting the social and emotional support they need usually (52.4%) or always (24.4%). The majority also rated their quality of life as good (33.5%) or very good (59.7%). There was wide variability in the reporting of finding comfort in religion or spirituality, with more than one-third (39.3%) finding comfort most days, every day, or many times a day, almost one-third (32.4%) reporting some days, once in a while or never/almost never, and more than one-quarter (28.4%) reporting ‘not applicable’. Almost half (45.2%) reported having thought about or begun thinking about retiring from full-time employment, 19.6% reported being unsure, but likely within five years, and 24.7% were unsure, but likely within ten years. Ten percent (10.5%) reported that they had not thought about retirement. Among those who answered that they had thought about or begun thinking about retirement, the average age that they anticipated retiring was 67.8 (SD = 4.3).

Among those who answered the caregiving question (*n* = 2086), 396 (19.0%) reported providing care on an on-going basis to a family member, friend, or neighbor with a chronic illness or disability. This included 22.4% of the female faculty members and 17.3% of the male faculty members. Compared to those who reported having no caregiving responsibilities, the caregiving faculty members were proportionally (*p* < 0.05) more likely to be: female; younger (mean age 61.4 vs. 62.5); minority (8.1 vs. 4.6%) living in slightly larger households (40.0% of caregivers lived in households of 3 + people vs. 25.0% not caregiving); and less likely to be financially secure (63.0% of caregivers reported that their finances were secure vs. 73.0% of those not caregiving). Caregiving faculty members were also statistically less likely than their non-caregiving counterparts to report that: their health was excellent (37.7 vs. 45.4%); they always get the social and emotional support they need (16.1% vs. 26.2%); and that their quality of life was very good (49.1 vs. 62.1%). However, caregiving faculty members were more likely to report that they find comfort in their religion or spirituality many times a day or every day (32.6 vs. 24.2%) compared to their non-caregiving counterparts.

There were no statistically significant differences between caregivers and non-caregivers regarding thoughts about retirement; an equal proportion of caregivers and non-caregivers (45.4 and 45.6%, respectively), reported having thought about or begun thinking about retiring from full-time employment in academic medicine, at an average age of 67.8.

Among the caregivers (*n* = 396) who answered the caregiving strain question (*n* = 388), close to one-quarter (21.6%) reported that providing care was associated with a lot of mental or emotional strain; 68.6% reported some strain, and only 9.8% reported no strain. Comparing male and female caregivers, approximately equal proportions of males and females reported similar levels of strain; that is, there were no statistically significant differences in caregiving strain by sex. Male caregivers were older than female caregivers (62.3 vs. 60.0), more likely to be married (95.4 vs. 72.8%), and lived in slightly larger households (2.6 mean number of people in the household vs. 2.3). The responses to self-reported health differed slightly by sex. Male caregivers reported higher rates of very good health (42.8 vs. 34.9%) and fair or poor health (6.4 vs. 2.7%) compared to female caregivers. Male caregivers also reported somewhat higher rates of very good quality of life compared to female caregivers (51.5 vs. 45.3%). There were no statistically significant differences by sex among the caregivers in personal finances, depression, social support, religiosity, or thoughts about retirement. However, among those who reported that they had thought about or begun thinking about retirement, male caregivers indicated retiring at an older age (68.7), on average, compared to female caregivers (66.2).

## Discussion

We found high rates of both caregiving and caregiving-related strain among late-career faculty members in medical schools. In this report, we found that nearly one-fifth (19%) of the full-time faculty members age 55 or older reported providing care for someone with a chronic illness or disability on an on-going basis. This 19% overall rate of caregiving is higher than the overall national average of 16% [11] and the 12% rates found in previous national epidemiologic surveys that used the same caregiving status question [14, 24]. We also found that 90% percent of the caregivers reported experiencing some or a lot of mental or emotional strain providing care; only 10% reported no strain. This rate of some or a lot of caregiving strain is also higher than the reported rates (67% and 71%, respectively) in two previous national epidemiologic surveys that also used the same caregiving strain question [14, 24]. Thus, participants in our current sample reported both higher rates of caregiving and more caregiving strain than previous studies of comparably aged caregivers.

Furthermore, in addition to finding that 22.4% of female faculty members were serving in a caregiving role, we also found a relatively high rate of male caregiving; 17.3% of the male faculty members reported providing caregiving, which aligns with Wolff et al.’s findings that that men are increasingly serving in caregiver roles [17].

Regarding health and well-being, we found that relative to their non-caregiving counterparts, caregiving faculty members were less likely to report: excellent health; adequate social and emotional support; and very good quality of life. In secondary analyses (not reported here), we also found that the faculty caregivers who reported a lot of strain were nearly twice as likely to report depression as caregivers who reported only some strain and that more caregiver strain was also inversely associated with happiness, social and emotional support, good quality of life.

Finally, we also observed that caregivers in our study were statistically more likely to report finding comfort in their religion or spirituality every day compared to their non-caregiving peers. There was a similar trend for reporting every day comfort in religion or spirituality among the no strain group, although not statistically significant. Although there is no literature directly addressing the association between caregiving and religion or spirituality as it relates to late-career experiences of faculty members, there is literature showing association between daily spiritual experiences and better self-rated health and social networks [29] and a documented association between religiosity/spirituality and life satisfaction [30]. Further exploration of this topic may generate innovative faculty development interventions around caregiving, social support, and coping.

High rates of both caregiving and caregiving strain, as well as the diminishing socially constructed gender roles in caregiving point to opportunity for deliberative, and strategic planning around policies, programs, and resources to mitigate deleterious impacts of caregiving in late-career. Human resource offices, wellness programs, and faculty development offices should collaborate to address current and future employee caregiving–work role conflicts and responsibilities and institutional workforce strategies.

### Policies, programs, and resources

Many of our institutions already offer a variety of resources and there are myriad programs in the community; for example: respite care; sitter/companion programs; home health/visiting nurses; meal preparation; shopping services; chore/task services; financial planning; caregiver-focused web-based interventions [31]; and online/virtual support groups (e.g., Facebook, Instagram, Twitter). However, there is little evidence documenting the utilization and impact of these programs on faculty outcome measures, such as well-being and burnout or institutional outcomes, such as recruitment, retention, faculty satisfaction, promotion, or retirement.

Clearly, the increased prevalence of caregiving and caregiving strain is not limited to aging faculty members, nor to faculty working in academic medicine. Faculty members starting families and those who have young children are well-versed in childcare challenges. In fact, there is at least one national effort directed towards early-career faculty members who have caregiving challenges. For example, the Doris Duke Foundation [32] has awarded ten U.S. medical schools with award funding to provide supplemental, flexible funds to early-career physician scientists who face extraprofessional caregiving demands. The goal of the program is to retain early-career physician scientists. To our knowledge, there are no similar national or institutional efforts to retain mid- or late-career faculty who also face extraprofessional caregiving demands.

Although our institution does not collect data from our employees about their general childcare or eldercare responsibilities, our institution does track utilization of caregiving benefits. For example, among our institution’s full-time faculty age 55 and older in the school of medicine, 2.7% accessed caregiving benefits in the 2020 calendar year compared to 8.9% of their faculty peers younger than age 55. Institutions may begin to understand caregiving challenges among their late-career faculty by exploring those who access caregiving or related benefits to ascertain needs, gaps in service, and impact on career and retirement planning.

Our findings also illuminate the necessity for more in-depth investigation of older faculty members’ needs, particularly as these needs impact work performance or as the needs either hasten or impede career transition decisions, including retirement. For example, disproportionately, in our survey, respondents less than age 65 were more likely to be caregivers than those age 65 and older; 72% of the male caregivers were younger than age 65 and 92% of the female caregivers were younger than age 65. It may be that faculty members who are older than age 65 are less likely to be providing care for someone else because the care recipients are more likely to be residing in a care facility or because the caregivers themselves are unable to provide physical care for someone else. It is uncertain how caregiving responsibilities impact retirement decisions. In this work, we did not find a statistical difference between caregivers and non-caregivers regarding their plans for or expected age at retirement; nevertheless, transparent, institutionally supported opportunities and resources would likely have positive benefits for both employees and their employers in retirement planning. Our findings amplify significant gaps in the literature for how late-career faculty members navigate the caregiving–career–retirement overlap and transitions; what does exist is efforts undertaken in select groups of faculty members and at individual institutions [33, 34].

Retirement programming is important not only for faculty members, but also for institutions. Naturally, older faculty members’ salaries are higher than their junior and mid-career faculty counterparts and institutional financial models can demonstrate cost-savings through retirement. However, some faculty members do not believe that they can comfortably retire, or do not wish to retire. Our data show that approximately three-quarters (71%) of the population felt that their personal finances were sufficient, but 12% said not sufficient and 17% were not sure. Furthermore, 16.5% of caregivers reported that their personal finances were not sufficient compared to 10.7% among non-caregivers and 21% of caregivers were unsure about their personal finances, compared to 16% of their non-caregiving peers.

Given the assumption that medical school faculty are economically privileged, what is the origin of this perception of financial insufficiency or financial uncertainty? Does it reflect inadequate savings, increased caregiving-related expenses, or reduced earnings? Additionally, how does caregiving and the availability of caregiving programming and resources, including those addressing the well-being of caregivers, parlay into retirement decision-making? Currently, we do not know how caregiving responsibilities at home intersect and overlap with work responsibilities for late-career academic faculty.

## Limitations

There are several limitations to this study. First, our findings are from a convenience sample of faculty from only 14 of the 145 LCME-accredited U.S. medical schools and may not be generalizable to faculty at any individual medical or other school of higher education. Nonetheless, it should be noted that the 14 schools surveyed were purposely selected to maximize representativeness of the US LCME-accredited medical school population based on a number of factors. Second, these cross-sectional data were collected during a snapshot in time in 2017, and the response rate was only 41%. Third, aiming for questionnaire brevity, the well-being, caregiving, and caregiving strain items were all single-item, self-report.

Another limitation of this project is that because the parent study was not primarily focused on caregiving, we did not examine the nature or intensity of the faculty members’ caregiving experiences, which is very important. For example, in their recent analysis of national trends in family caregiving between 1999 and 2015, Wolff et al. found that the primary caregivers were overwhelmingly spouses and adult children [17]. They observed that caregiving arrangements lasted four years or longer on average and that primary caregivers provided approximately 30 h of care per week. Although untested, we may hypothesize that faculty members’ caregiving efforts are equally significant. Similarly, we did not examine the association of the caregiving experience with the work experience, other than correlating caregiving with thinking about retirement. For example, in their systematic review of the international research on unpaid caregivers and labor market choices, Lilly, Laporte, and Coyte found that ‘heavily involved/intensive’ caregivers were *more* likely to withdraw from the labor market than their counterparts [35]. Although the data vary, in general, most studies have shown a moderate reduction in the number of hours worked per week among caregivers and an inverse association between caregiving intensity and hours worked per week. Finally, we did not collect any data from or about survey non-responders; thus, we do not know the status of caregiving or caregiving burden among those who did not participate in the survey.

## Conclusion

Our data highlight the caregiving responsibilities and concomitant mental or emotional strain and well-being of a significant proportion of the U.S. medical schools’ rapidly aging workforce. Aging and caregiving are universal and cross-cutting realities. Future lines of inquiry that impact both faculty members and leaders in human resources and faculty development may include: (1) are older caregiving faculty members with increasing caregiving strain less likely to: submit grant applications; conduct research; spend time in clinic; spend time mentoring junior faculty members? (2) Are older caregiving faculty members with increasing strain more likely to: retire; have worse mental or physical health; abuse substances; behave badly toward colleagues, trainees, or patients; make more clinical errors? (3) Do older caregiving faculty members have unique needs that differ by sex? These questions and more require further thought and exploration to both maximize employee productivity, satisfaction, as well as workforce planning.

Regardless of industry, employers are likely recognizing, or will soon recognize, the increasing age of their employees, the resulting pull of caregiving responsibilities and associated effects, and how these factors weigh on workforce and retirement planning best practices. We in faculty affairs, faculty development, and human resources should increase our attention to these expanding needs by developing appropriate policies, programs, and resources.

## Data Availability

The data that support the findings of this study are available from the Association of American Medical Colleges (AAMC) but restrictions apply to the availability of these data, which were used under license for the current study, and so are not publicly available. Data are, however, available from the authors upon reasonable request and with permission of the AAMC.

## Abbreviations

AAMC: Association of the American Medical Colleges
LCME: Liaison Committee on Medical Education
SPSS: Statistical Package for the Social Sciences
U.S.: United States
